# Implementing and Integrating a Clinically Driven Electronic Medical Record for Radiation Oncology in a Large Medical Enterprise

**DOI:** 10.3389/fonc.2013.00069

**Published:** 2013-04-18

**Authors:** John P. Kirkpatrick, Kim L. Light, Robyn M. Walker, Debra L. Georgas, Phillip A. Antoine, Robert W. Clough, Heidi B. Cozart, Fang-Fang Yin, Sua Yoo, Christopher G. Willett

**Affiliations:** ^1^Department of Radiation Oncology, Duke UniversityDurham, NC, USA; ^2^Duke Health Technology Solutions, Duke UniversityDurham, NC, USA

**Keywords:** electronic medical record, electronic health record, quality assurance, patient safety, radiation oncology practice, charts, information technology

## Abstract

**Purpose/Objective**: While our department is heavily invested in computer-based treatment planning, we historically relied on paper-based charts for management of Radiation Oncology patients. In early 2009, we initiated the process of conversion to an electronic medical record (EMR) eliminating the need for paper charts. Key goals included the ability to readily access information wherever and whenever needed, without compromising safety, treatment quality, confidentiality, or productivity.

**Methodology**: In February, 2009, we formed a multi-disciplinary team of Radiation Oncology physicians, nurses, therapists, administrators, physicists/dosimetrists, and information technology (IT) specialists, along with staff from the Duke Health System IT department. The team identified all existing processes and associated information/reports, established the framework for the EMR system and generated, tested and implemented specific EMR processes.

**Results**: Two broad classes of information were identified: information which must be readily accessed by anyone in the health system versus that used solely within the Radiation Oncology department. Examples of the former are consultation reports, weekly treatment check notes, and treatment summaries; the latter includes treatment plans, daily therapy records, and quality assurance reports. To manage the former, we utilized the enterprise-wide system, which required an intensive effort to design and implement procedures to export information from Radiation Oncology into that system. To manage “Radiation Oncology” data, we used our existing system (ARIA, Varian Medical Systems.) The ability to access both systems simultaneously from a single workstation (WS) was essential, requiring new WS and modified software. As of January, 2010, all new treatments were managed solely with an EMR. We find that an EMR makes information more widely accessible and does not compromise patient safety, treatment quality, or confidentiality. However, compared to paper charts, time required by clinicians to access/enter patient information has substantially increased. While productivity is improving with experience, substantial growth will require better integration of the system components, decreased access times, and improved user interfaces. $127K was spent on new hardware and software; elimination of paper yields projected savings of $21K/year. One year after conversion to an EMR, more than 90% of department staff favored the EMR over the previous paper charts.

**Conclusion**: Successful implementation of a Radiation Oncology EMR required not only the effort and commitment of all functions of the department, but support from senior health system management, corporate IT, and vendors. Realization of the full benefits of an EMR will require experience, faster/better integrated software, and continual improvement in underlying clinical processes.

## Introduction

As a medical specialty, radiation oncology relies heavily on technology for planning and treating cancer patients. While a variety of information and practice management systems are available (e.g., ARIA from Varian Medical Systems, Palo Alto, CA, USA and Mosaiq from Elekta, Stockholm, Sweden), these systems are specific to radiation oncology and are quite difficult to integrate with enterprise-wide information systems. Thus, radiation oncology departments have typically generated and utilized department-specific paper charts, containing planning and treatment data unique to radiation oncology, as well as demographic and disease-specific information for a particular patient. As a result, much of the information in the radiation oncology chart was essentially inaccessible to other practitioners in the health system or duplicated information in the enterprise-wide medical system and the planning/treatment data base. As an example, the historical organization of information in our department is shown in Table 1.

**Table 1:** **Distribution of patient/treatment information in our department pre-implementation of a radiation oncology EMR**.

RadOnc paper chart	Enterprise-wide EMR	ARIA/eclipse
**Consult note**	**Consult note**	Planning orders
**RT prescription**	Lab reports	**RT prescription**
Treatment plans	Radiology reports	Treatment plans
**Path reports**	**Path reports**	Daily treatment log
**Treatment summary**	**Treatment summary**	QA reports
Weekly notes	Follow-up notes	Task pad
**Consents**	**Consents**	RadOnc schedules
Vitals and some medications	Medications and some vitals	

*Bold face indicates documents that appear in multiple information systems*.

In 2007, we recognized that an electronic medical record (EMR) could offer an improved ability to distribute and access radiation oncology planning, treatment, and patient management information throughout our enterprise. In addition, we saw potential benefits within the department from an EMR, including improved access to information, i.e., no more need to locate the paper chart, and decreased expenses associated with creation, storage, and retrieval of paper charts. At the same time, we appreciated that the paper chart offered many desirable attributes, including ready access to radiation oncology planning/treatment information in a single place and a high level of comfort with its use on the part of radiation oncology providers. Nonetheless, we concluded that an EMR was inevitable and potentially a significant advance.

This paper presents our experience with development of a radiation oncology EMR in the Duke University Health System, an enterprise that is composed of over 1,500 practitioners with more than two million patient visits annually.

## Materials and Methods

In developing the goals of the goals and objectives for a radiation oncology EMR, departmental, and health system leadership agreed that the EMR must have the attributes shown in Table 2.

**Table 2:** **Required attributes for the radiation oncology EMR**.

Does not compromise safe/effective treatment of patients
Ensures integrity of patient data
Complies with government/institution requirements for documentation and billing
HIPAA compliant
Facilitates communication internally and externally
EMR is accessible to whomever needs this information at all times
Requires less net effort than existing paper chart
Utilizes existing ARIA and enterprise-wide internet/information system
No major software/hardware purchases

It was clear that we must continue to provide safe and effective treatment of our patients, and, in doing so, that we comply with all regulatory policies and principles of good medical practice. The EMR must also facilitate communication within the department and throughout the organization. As one senior thoracic surgeon stated, “When a patient receiving radiation for lung cancer shows up in the emergency department in the middle of the night, I need to know right then and there what you [Radiation Oncology] have been doing to him.” Thus, the information in the EMR would have to be immediately and easily accessible to whomever needs it, wherever and whenever necessary. As discussed below, this required that we identify what information is needed by whom.

While we recognized that work flow would change with implementation of an EMR, we established an objective that the EMR should not require a net effort greater than that of the existing process. Finally, the project was constrained by the requirement that we utilize the existing ARIA radiation oncology and enterprise-wide information technology (IT) infrastructure without significant expenditures.

To meet the above ambitious goals, we established a formal project guided by the following set of principles (Table 3).

**Table 3:** **EMR project principles**.

Start with existing chart and most important processes
Replace current processes with “electronic version”
BUT, automate data entry and transfer where possible
Shoot for workable (as opposed to perfect) solution
Test as we go along
Processes will be “designed/built” through EMR team at central campus
Communicate to department continually
Create buy-in
BUT, no one allowed to “opt out” of EMR
Establish firm deadline for conversion to EMR

While the above list appears straightforward, a great deal of debate went into its creation. At the outset of this, some argued that we start with a “blank slate,” i.e., design an ideal information system and then build and/or select the system that best meets those specifications. However, given the constraints that we utilize existing systems, minimize expenditures, and meet deadline for implantation, we instead chose to strive for a “less-than-perfect” solution that was workable and implementable. Specifically, we elected *not* to design a system which automatically stores all patient and treatment-related data in a readily queryable database, facilitating our research mission.

In order to effectively implement the EMR, it was clear that we needed to create consensus from all stakeholders. To do so, multiple discussions and meetings were held throughout the department. Nonetheless, several senior staff argued that establishing an EMR was unnecessary, wasteful of time and money, a diversion of attention from patient care, and/or of unproven benefit. Despite these objections and a suspicion on many members of the department that “some people will never use an EMR,” senior leadership unambiguously communicated that there was no option to “opt out” of using an EMR after it was established.

The importance of setting an achievable, but firm, deadline for conversion to an EMR at the outset of the project is paramount. Once in place, the sanctity of this deadline must be reinforced at all levels continually, and project milestones created and progress measured against this deadline.

Given the broad impact of an EMR, we created the following project team composed of all departmental stakeholders, as well as a representative from the health system IT group (Table 4).

**Table 4:** **Composition of radiation oncology EMR team**.

Departmental administration
Administrative assistants, medical records, schedulers, operations management
Departmental information technology
Dosimetry
Hospital IT
Nursing
Physicians
Physics
Radiation therapists

The team met weekly or biweekly, depending on the tasks at hand, and was led by the Clinical Director and the Chair of the department. Prior to each meeting a detailed agenda was distributed, along with minutes from the previous meeting. These minutes, which were generated by a dedicated scribe, contained a point-by-point summary of items discussed at the meeting along with action items specifying timing and responsibility.

In addition to the team members, it was also essential to secure the commitment of senior hospital administration and our radiation oncology software vendor (Varian) to the success of this project.

## Results

The EMR project was initiated in February, 2008, with a deadline for implementation of January, 2009. On January 4, 2009, the department converted from paper charts to the EMR.

To meet the specifications, a virtual radiation oncology chart was designed, composed of two primary systems (Table 5 below).

**Table 5:** **Virtual radOnc chart: ARIA + eBrowser**.

**Utilize ARIA for radiation oncology-specific processes, e.g**.
Treatment planning orders
Radiation therapy prescription
Workflow management
Treatment plan with approvals
Characterized by specialized information neededonly within RadOnc
**Utilize eBrowser for all other info, e.g**.
Consult notes, treatment summaries
Weekly treatment check notes, nursing notes
Treatment planning/simulation notes
Consents, patient intake forms
Selected outside data
Characterized by any information needed outside of RadOnc

Information that would be utilized only in radiation oncology, e.g., treatment planning orders, dose distributions, etc., would be placed in ARIA. In contrast, any information that would be potentially needed outside of Radiation Oncology, e.g., consultation notes, weekly on-treatment visit notes, consents for treatment, would reside in the enterprise-wide information system eBrowser (McKesson, San Francisco, CA, USA).

Management of external documents was a major issue in developing the above system. With a paper chart, external documents, such as outside pathology, radiology, and operative reports, were filed in the radiation oncology chart. After extensive discussions, the hospital leadership approved scanning these documents and internal hand-completed forms, such as patient intake questionnaires and treatment consents, into eBrowser. Access to these documents was substantially facilitated by placing them with clear titles in eBrowser, e.g., “External Radiology Report.”

The detailed location of specific documents in the EMR is shown in Table 6.

**Table 6:** **Location of specific documents in the virtual radiation oncology chart**.

**ARIA**	**eBrowser**
Treatment planning orders	Consultation notes*
Radiation therapy prescription	RadOnc weekly on-treatment notes*
Treatment record	Treatment planning/sim note*
Radiation therapy notes	Nursing notes*
Planning/treatment tasks	Treatment summary*
QA documentation	Patient intake questionnaire**
On-treatment images	Consents**
2D/electron calculations	Internal lab, pathology, radiology, procedure reports*
**Eclipse**	External documents**
Contours	**PACS**
Treatment plans (dose distribution/DVH)	Diagnostic radiology images

*Directly entered into eBrowser; **scanned into eBrowser.

Utilizing the EMR required installation of patient information network (PIN) workstations in every exam room and physician work room to provide ready provider access to eBrowser. In addition, working with Varian, we installed access to ARIA/Eclipse via a Citrix-server on the PIN workstations. Thus, providers have the full capabilities of the virtual radiation oncology chart from any PIN workstation.

**Figure 1:**
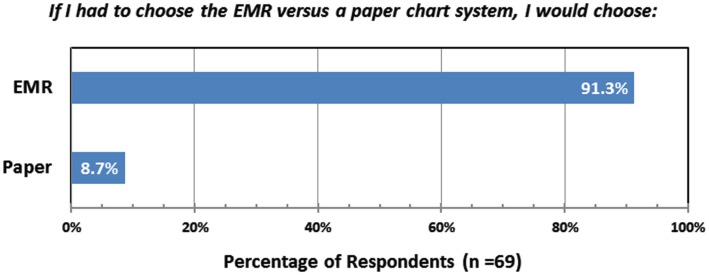
**Responses from a web-based survey of the Duke Radiation Oncology Department, conducted 1 year after conversion from paper charts to the EMR**.

One year after implementation of the radiation oncology EMR, a web-based survey of the department revealed a high level of satisfaction with the EMR (Table 7). Of the 70 respondents, 81 and 78% agreed that the EMR had improved the quality of patient care and patient safety, respectively. Ninety percent of respondents agreed that EMR improved the quality of medical records versus paper charts while only 25% agreed that information was harder to find in the medical record. However, only 34% of those surveyed agreed that they got the same amount of work done in less time with the EMR. When asked if they had to choose between the EMR and a paper chart, which they would select, 63 of 69 respondents (91%) chose the EMR (Figure 1).

**Table 7:** **Results of a web-based survey of the Duke radiation oncology department on the EMR, conducted 1 year after conversion from paper charts to the EMR**.

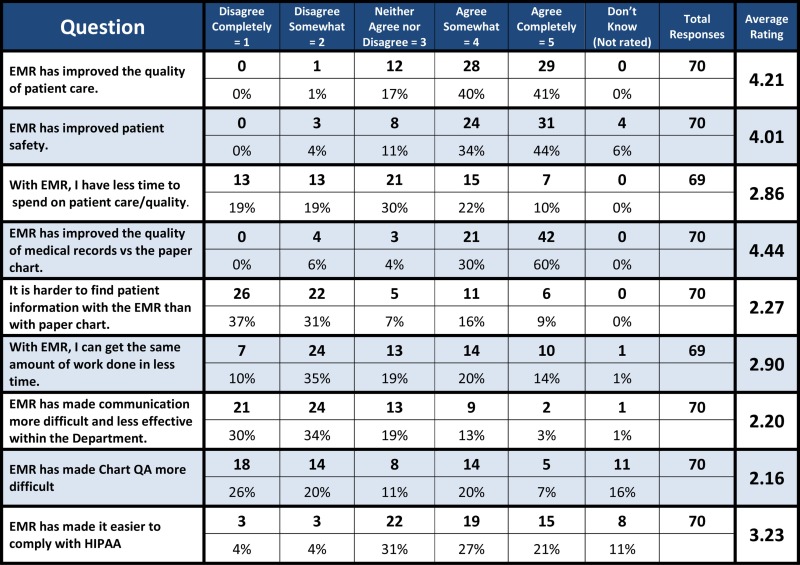

Though practitioners out side the department were not queried using a questionnaire, we rarely, if ever, receive complaints about a lack of information on a patient’s plan for or progress during a course of radiation therapy No significant safety or quality problems attributable to the EMR have been observed. Between 2008 and 2010, the rate of treatment deviations (as a percentage of patients treated) fell from 0.24 to 0.12%.

## Discussion

Timely, appropriate and clear communication and documentation is a fundamental requirement of radiation oncology practice (Martin, 1999; Bria II and Shabot, 2005; IMRT Documentation Working Group et al., 2009; Bleich and Slack, 2010; Moran et al., 2011; American Society for Radiation Oncology, 2012; Chera et al., 2012). An EMR offers the promise of improving the effectiveness and efficiency of communications, documentation, work flow, and ultimately, patient care (Salenius et al., 1992; Sailer et al., 1997; Law, 2005; Middleton et al., 2009). Moreover, an EMR will essentially be required in the United States in order to meet the requirements of various federal initiatives, avoiding penalties for non-compliance (Centers for Medicare & Medicaid Services, and HHS, 2012; Office of the National Coordinator for Health Information Technology, 2012; Shen et al., 2012). Of course, while an EMR can be a valuable component of a radiation oncology’s quality and safety program, other aspects of the program are at least as important, including establishing a culture that fosters safety and quality, exercising leadership on these issues, and utilizing the optimum combination of new technology, tried-and-true clinical practices, and commonsense (Bleich and Slack, 2010; Marks et al., 2011; American Society for Radiation Oncology, 2012; Chera et al., 2012; Pawlicki et al., 2012).

Implementation of the EMR in our department was accomplished on-time without compromising patient safety or treatment quality. While the capital outlay for new hardware and software modifications was minimal ($127,000), we estimate that over 1,000 h of effort was expended in designing, executing, and implementing this project – much of it from radiation oncology clinic staff (Table 8). These costs are partially offset by a reduction in annual costs of paper and supplies to create these charts (∼$21,000/year.) In addition, there are less well-quantified savings associated with the reduced need to file charts and newly received documents, balanced by the cost of scanning documents into the enterprise-wide system. The EMR clearly eliminates the problem of “misplaced” charts and provides ready access to all information, but this access can be hampered by the need for multiple log-ins (common), slow hardware/software/internet speeds (variable severity and intermittent), and system outages (rare).

**Table 8:** **The “balance sheet” for our radiation oncology EMR**.

Plus	Minus
Annual paper/supply savings: $21K	One-time capital costs: $127K + 1000’s of man-hours invested
No lost charts	Hardware/software downtime
Information accessible anywhere, anytime	Slow information retrieval
Filing eliminated	Software not fully compatible
Compliant with policies, safe practice	Learning curve (slightly) painful
Disruptive	Disruptive

Designing/selecting and implementing a radiation oncology EMR that is compatible with a health system’s information system is a disruptive process. Clearly, the disruption of existing practices has cost associated with changing practices, new infrastructure, and the replacement of the familiar paper charts. If conversion to an EMR is poorly executed (or communicated), this disruption can lead to degraded quality or unsafe conditions. On the other hand, disruption in this situation can have long-term benefits if it forces us to question our existing practices, consider the unfamiliar and adopt improved practices, procedures, and technologies. Finally, the most meaningful question is whether an “EMR helps deliver better quality and/or more cost effective health care.” This is an important, difficult question and, to our knowledge, there is not yet an objective answer (Menachemi and Collum, 2011).

## Conclusion

Successful and safe implementation of a Radiation Oncology EMR can be accomplished with minimal capital costs in a reasonable time-frame given complete commitment and substantial effort from the department and the support of senior management, the enterprise-wide IT department, and information system vendors. Full realization of the clinical benefits of an EMR will require experience, faster/better integrated software, continual improvement in underlying clinical processes and objective analysis of costs and savings. We believe that the structure for the Radiation Oncology EMR described in this paper is compatible and be integrated with the electronic health record system (Epic, Verona, WI, USA) being adopted by our health system.

## Conflict of Interest Statement

The authors declare that the research was conducted in the absence of any commercial or financial relationships that could be construed as a potential conflict of interest.
